# Visual hallucinations associated with varenicline: a case report

**DOI:** 10.1186/1752-1947-3-7560

**Published:** 2009-05-08

**Authors:** B Mahendri Raidoo, Eric C Kutscher

**Affiliations:** 1 Department of Psychiatry, Sanford School of Medicine, The University of South Dakota, Sioux Falls, SD 57108, USA

## Abstract

**Introduction:**

Varenicline is widely used for smoking cessation. It has shown efficacy over placebo and bupropion in manufacturer-sponsored trials. Those with mental illness were excluded from these trials. There are case reports of exacerbation of mental illness and development of psychiatric symptoms with varenicline use.

**Case presentation:**

A 61-year-old male Caucasian being treated for post-traumatic stress disorder, depression not otherwise specified and alcohol dependence, was prescribed varenicline while he was in a post-traumatic stress disorder/alcohol dual diagnosis treatment program. He developed visual hallucinations, which became worse with titration of the medication. These symptoms resolved upon discontinuation of varenicline.

**Conclusion:**

Patients with mental illness have a higher incidence of nicotine dependence, and attempts should be made for smoking cessation. Varenicline has not been widely tested in this population. There are reports of exacerbation of mental illness, and probable causation of psychiatric symptoms in the mentally ill. Providers should be aware of this possibility and advise their patients appropriately.

## Introduction

The FDA approved varenicline in 2006 as an aid to smoking cessation. It provides a unique mechanism compared to nicotine replacement therapy. It is a partial agonist selective for the alpha 4 beta 2 nicotinic acetylcholine receptor subtype. It provides a low to moderate level of dopamine stimulation, which is believed to alleviate craving and nicotine withdrawal symptoms. Additionally, this medication is an antagonist at nicotine receptors, which may reduce the reinforcing effects of nicotine and decrease the risk of relapse [1].

Psychiatric side effects are listed as a rare occurrence in the product information. Hallucinations, bradyphrenia, euphoric mood, psychotic disorders and suicidal ideation are listed as rare treatment emergent events [2]. Case reports have been published highlighting the potential for serious psychiatric side effects of this medication (Table 1) [3-5]. Jorenby *et al.*[6] reported acute psychosis, and emotional lability as serious adverse effects that occurred in a patient during treatment with varenicline, and continued in the patient following discontinuation of treatment. The nature of the psychosis is not described. In November 2007, the FDA issued a safety alert to healthcare professionals regarding reports of suicidal thoughts, and aggressive and erratic behavior in patients on varenicline. In May 2008, the FDA advised that prescribing information for varenicline was revised to include information on serious neuropsychiatric symptoms in the WARNINGS and PRECAUTIONS sections of the label [7].

**Table 1:** Case reports

Author	Patient gender and age	Pre-existing diagnosis	Symptoms with varenicline use
Freedman [3]	Female, 42	Schizophrenia	5-day psychotic episode
Kohen and Kremen [4]	Male, 63	Bipolar disorder	Manic episode
Morstad *et al.*[5]	Female, 41	Bipolar Disorder Type II and polysubstance abuse	Hypomania with agitation

Subsequently, a report by Moore *et al.*[8] for The Institute for Safe Medication Practices expressed immediate safety concerns about the use of varenicline in patients in whom lapses of alertness or motor control could lead to massive serious injury. They recommended caution with varenicline use, and consideration of alternative methods of smoking cessation. They also urged the FDA and the manufacturer to provide appropriate warnings, and to undertake further investigation. The report states that between May 2006 and December 2007, 55 cases of hallucinations were reported. It is not clear whether visual hallucinations were reported amongst these.

Stapleton *et al.*[9] conducted a study with 412 participants at an NHS tobacco dependence clinic in London, UK, where none of the abovementioned exclusion criteria were used. Twenty seven percent of the patients reported they were being treated for mental illnesses including depression, bipolar disorder, psychosis, and eating disorders. The investigators reported no evidence of exacerbation of mental illness symptoms by varenicline at the manufacturer recommended schedule, nor any evidence that adverse symptoms were experienced more in those with mental illness. Interestingly though, one participant had a severe psychological reaction, likened to a "bad LSD trip", including anxiety, paranoia, confusion and impaired motor control, that was not explained further. Sustained release of dopamine is postulated to be a contributing factor to the development of symptoms of psychosis.

## Case presentation

A 61-year-old Caucasian male army veteran had been treated as an outpatient for the last 2 years for post-traumatic stress disorder (PTSD), alcohol dependence, and depression not otherwise specified (NOS). Before this, he had been symptomatic, but did not present for treatment. He was admitted to a residential PTSD/alcohol dual diagnosis treatment program. His medications upon admission were fluoxetine 20 mg every morning for depression, nortriptyline 25 mg at bedtime for sleep and depression, quetiapine 50 mg at bedtime and every 6 hours as needed for anxiety, prazosin 1 mg at bedtime for nightmares, pramipexole 0.5 mg at bedtime for restless legs syndrome, terazosin 5 mg at bedtime for benign prostatic hypertrophy, atenolol 50 mg daily, and spironolactone 50 mg daily, for hypertension. He had been on all these medications for at least 6 months, with no reported adverse effects. He had smoked 2 packs of cigarettes per day for the past year, and had previously smoked four packs per day, for about 30 years. The patient's diagnosis of restless legs syndrome was made before any treatment he received for mental illness, and was not thought to be a medication adverse effect. His laboratory studies were negative and he did not have anemia (his hemoglobin level was 13.5 just before his enrolment in the treatment program).

The patient ceased using alcohol 1 month before entering the treatment program. He denied using any alcohol throughout the duration of the residential program, and no such evidence was found to the contrary by his health care providers. Three weeks before completion of the program, he was prescribed varenicline for smoking cessation, at the manufacturer recommended titration. This was prescribed by a provider at the treatment program. Subsequently, the patient reduced his smoking to two cigarettes per day.

Upon starting varenicline, he reported experiencing visual hallucinations. This was unusual to him as he reports that he had never experienced visual or other hallucinations in the past. On increasing the medication to 1 mg twice daily, his visual hallucinations became more frequent and more vivid. He reported seeing ropes dangling in the air and birds flying around the room. Nineteen days after the commencement of varenicline, he was discharged to home. Initially, he had not reported his symptoms to his providers or family, fearing that they would think he was "crazy". On the trip home, his wife noticed his behavior had changed. She reported that on the drive home he was leaning over as though avoiding something and it appeared to his wife that he was "seeing things". His wife reported that he was also reaching out in the air as if to grasp something.

Upon arrival at home, he read through the drug information provided by the pharmacy and noted that hallucinations could be a side effect. He immediately discontinued varenicline, without consulting his physician, and the hallucinations reduced, and resolved over a period of 3 days. He reported these symptoms to his regular mental health physician provider at his next office visit. According to DSM-IV-TR criteria, a diagnosis of Psychotic Disorder Not Otherwise Specified would be appropriate [10]. No further investigations were performed at this time as he was asymptomatic.

On applying the Naranjo causality scale, a score of 6 was obtained, indicating a probable adverse drug reaction to varenicline [11]. It is important to note that we took into consideration that this patient was on pramipexole, a dopamine agonist, which could have contributed to his symptoms. However, the patient had been using this medication for many months and had not reported signs or symptoms of psychosis.

## Discussion

Nicotine is believed to act through the neurobiological reward pathways in the brain, most notably involving dopamine, but also other neurotransmitters. Smoking tobacco stimulates a rapid rise in dopamine in the nucleus accumbens, which is believed to contribute to the addictive properties of nicotine [1]. Tolerance may develop in heavy smokers, resulting in increased use, and dependence.

Attempts at smoking cessation may be difficult due to nicotine withdrawal symptoms. Criteria for nicotine withdrawal include dysphoric mood, insomnia, irritability, frustration or anger, anxiety, difficulty concentrating, restlessness, decreased heart rate, and increased appetite or weight gain [10]. Nicotine withdrawal symptoms are expected to be of short duration, and to improve with time. Visual hallucinations have not, to our knowledge, been described in the literature as a symptom of nicotine withdrawal.

Nicotine receptor partial agonists, such as varenicline, may help smoking cessation by selectively activating the alpha 4 beta 2 neuronal nicotinic acetylcholine receptors. These receptors modulate the effects of the mesolimbic dopamine pathways, which mimic nicotine, thus helping counteract withdrawal symptoms. These medications may also block the dopamine releasing effect of nicotine, thus reducing pleasure from smoking. Varenicline was developed from cytisine, a naturally occurring alkaloid compound, shown to be an effective partial agonist for the alpha 4 beta 2 receptor. Cytisine has been used in central and eastern Europe for smoking cessation for over 40 years [12]. A literature review and meta-analysis of research conducted on cytisine suggested that it is effective for smoking cessation. One of the published studies reviewed included patients with mental illness. They reported adverse effects, and toxic effects of cytisine, which did not include symptoms of psychosis [13].

Amongst the exclusion criteria for the phase three varenicline trials were major depressive disorder within the previous 12 months requiring treatment, history of or current panic disorder, psychosis, bipolar disorder or eating disorder; alcohol or drug abuse/dependency within the previous 12 months, as well as psychogenic medication use [14,15]. Psychiatric side effects are listed as a rare occurrence in the product information [2]. Hallucinations, bradyphrenia, euphoric mood, psychotic disorders and suicidal ideation are listed as rare treatment emergent events [2]. Further details of these adverse effects are not available in the published medical literature.

There are also reports of individuals with psychiatric disorders using varenicline without subsequent adverse effects. Fatemi reported a case of a patient with schizophrenia who reduced his tobacco use to five cigarettes per day with 24 months of varenicline utilization, who did not experience any exacerbation of his psychiatric symptoms [16]. Evins and Goff report a series of 19 psychiatrically stable patients with schizophrenia who did not experience a psychiatric re-hospitalization or exacerbation of their symptoms with 24 weeks of varenicline use [17]. Exact treatment recommendations cannot be drawn from these case reports as to the utility of varenicline in patients with psychiatric illnesses.

## Conclusion

We report a patient with visual hallucinations that developed with varenicline use. The patient had pre-existing mental illness. Many patients with mental illness are concurrent smokers and the use of this product has become very common in this population. At the writing of this paper, three cases have been published showing exacerbation of mental illness from varenicline (Table 1). The FDA and the Institute for Safe Medication Practices have advised providers of suicidal thoughts, depressed mood, aggressive and erratic behavior, and other neuropsychiatric symptoms with varenicline use. Patients with mental illness have a higher incidence of nicotine dependence, and attempts should be made at smoking cessation. Treatment should include behavioral and pharmacological strategies as necessary. Although patients with psychiatric illness have used varenicline with reported efficacy and tolerability, caution needs to be used in this population. This case highlights the need for providers to be aware of potential psychiatric symptoms with varenicline use. Providers should prescribe with caution and provide relevant education and monitoring to patients with underlying mental illness.

## Consent

Written informed consent was obtained from the patient for publication of this case report. A copy of the written consent is available for review by the Editor-in-Chief of this journal.

## Competing interests

The authors declare that they have no competing interests.

## Authors' contributions

BMR was the psychiatry resident involved in the care of the patient and the drafting of the manuscript. ECK was a major contributor in critical revision of the manuscript for important intellectual content. Both authors read and approved the final manuscript.
